# Environmental chemistry and ecotoxicology: in greater demand than ever

**DOI:** 10.1186/s12302-016-0101-x

**Published:** 2017-01-17

**Authors:** Martin Scheringer

**Affiliations:** 1 ETH Zürich, Institute for Chemical and Bioengineering, 8093 Zurich, Switzerland; 2 Masaryk University, RECETOX, 625 00 Brno, Czech Republic

**Keywords:** Environmental chemistry, Ecotoxicology, Risk assessment, Hazard assessment

## Abstract

Environmental chemistry and ecotoxicology have been losing support, resources, and recognition at universities for many years. What are the possible causes of this process? A first problem may be that the need for research and teaching in environmental chemistry and ecotoxicology is no longer seen because chemical pollution problems are considered as largely solved. Second, environmental chemistry and ecotoxicology may be seen as fields dominated by routine work and where there are not many interesting research questions left. A third part of the problem may be that other environmental impacts such as climate change are given higher priority than chemical pollution problems. Here, several cases are presented that illustrate the great demand for innovative research and teaching in environmental chemistry and ecotoxicology. It is crucial that environmental chemistry and ecotoxicology are rooted in academic science and are provided with sufficient equipment, resources, and prospects for development.

## Environmental chemistry and ecotoxicology under pressure

The publication of *Silent Spring* in 1962 [1] made the problem of chemical pollution broadly visible and initiated a political and scientific development that has shaped environmental chemistry and ecotoxicology as we know them. Since 1962, a lot of progress has been made, many important insights have been gained, and new methods have been developed. The objective of this paper is not to provide a critical review of the development over the last decades, but to analyze the current situation, the standing of environmental chemistry and ecotoxicology in the academic system with a focus on Germany and Switzerland. The result from this analysis is that the relevance and reputation of environmental chemistry and ecotoxicology in the academic system have been decreasing for years and also today, in 2016, the prospects are not good.

It is not for the first time that this concern is raised. In 2008, A. Schäffer, M. Roß-Nickoll, H.T. Ratte, and H. Hollert, all at RWTH Aachen, initiated UFOH, an association of university institutes active in environmental research and teaching. The goal of UFOH was to analyze both the status quo of chemical-related environmental research at universities and the prospects for its future development. In 2009, the members of this group stated [2]:

“Although qualified young environmental scientists are in great demand by industry and authorities, the number of university chairs in this field is steadily and disproportionately declining. Also, the financial support for research projects has been significantly shortened, unlike in other research areas, such as biotechnology or nanotechnology. (…) We are more than concerned that, in the future, both research and education will severely suffer with the ongoing budget reductions in environmental sciences at universities.”

Since then, this trend has been exacerbated. Recent examples from Switzerland include the following: after many years of successful and important work in the field of environmental organic trace analysis, the analytical chemistry group at EMPA has been reshaped and given a different focus; at the Department of Chemistry and Applied Biosciences of ETH Zurich, the Safety & Environmental Technology Group, where I have worked for 20 years, will be closed down in 2018 without a continuation; and, in 2015, the Swiss Society for Food Chemistry and Environmental Chemistry dropped the “Environmental” from its name and is now called Swiss Society for Food Chemistry [3].

Discussions with journalists and science writers seem to echo the lack of interest in chemicals, environment, and health. “Chemicals” as a topic is seen as too abstract and unwieldy; in science writing for newspapers and magazines, chemicals are frequently presented as an—important—element of other topics such as climate change or bee decline, but it is not often that chemicals as such are the main topic of a report.

Among industry, government authorities, and universities, industry appears to retain the importance of environmental chemistry and ecotoxicology. Obviously, this is because there is an immediate need for well-trained scientific and technical experts who work on the characterization and assessment of chemicals as an essential contribution to the registration of chemical products. In government authorities, the situation is mixed. In chemical-related units, the importance of environmental chemistry and ecotoxicology is fully acknowledged, but in other units, chemical-related work is often seen as a routine process in a highly regulated and clearly structured field without any open questions. In the universities, the situation is most difficult because here environmental chemistry and ecotoxicology are often seen as rather traditional or even outdated fields and priority is given to other, apparently more innovative, and more timely topics.

What are the root causes of the reservations, skepticism, and lack of support that environmental chemistry and ecotoxicology meet within universities? Three possible explanations are as follows:

- environmental chemistry and ecotoxicology are no longer needed because chemical-related problems have been solved to a large extent (“no need”);
- environmental chemistry and ecotoxicology are no longer vital and productive as academic subjects because they do not offer any interesting and novel research questions (“boring”); and
- environmental chemistry and ecotoxicology may be relevant and interesting, but other environmental problems such as climate change are more pressing and need to be given priority.

### Why environmental chemistry and ecotoxicology are in great demand

The examples presented below are two cases related to my own field of research, but there are many more cases that could be used to demonstrate the high demand for research and higher education in environmental chemistry and ecotoxicology.

### Example 1: polychlorinated biphenyls (PCBs)

The case of PCBs is particularly important and revealing because it concerns a subject that may be considered boring and outdated because so much research has been done on PCBs in the last decades. PCBs became a paradigmatic case of environmental contaminants when the paper by Jensen et al. [4], “DDT and PCB in marine animals from Swedish waters”, was published in *Nature*. But have the problems related to PCBs been solved? In 2016, almost 50 years later, Jepson and Law [5], in a paper in *Science*, call for more research into PCBs:

“In East Greenland polar bears, blubber PCBs increased unexpectedly between 2010 and 2013, resulting in PCB concentrations that were as high in 2013 as in 1983. (…) Future research should investigate pathways of PCB contamination of the marine environment.”

The problems caused by PCBs have not yet been solved. Surprisingly, even today, substantial PCB emissions take place [6], and, at the same time, it is not sufficiently clear what the sources of these emissions are. Government authorities assumed for more than 20 years that there were no relevant PCB emissions left after new production of PCBs had been banned in the 1980s in many countries, but this was not true. However, it took several years before our group at ETH Zurich was able to obtain funding for compiling an updated and more comprehensive PCB emission inventory for Switzerland (this project is currently ongoing).

Beyond the case of PCBs, the lesson learned from this example is that using highly persistent chemicals in numerous applications and products implies that research in environmental chemistry and ecotoxicology will be necessary for many decades. Importantly, also under REACH, many highly persistent chemicals have been registered and will be on the market for many years to come.

### Example 2: Incremental substitution and chemical property data under REACH

Under REACH, the European Chemicals Agency, ECHA, hosts a database that contains the various types of data submitted with the chemicals’ registration dossiers. The list of chemicals registered up to now and the chemical property data of these chemicals as they are presented in the ECHA database [7] highlight two problems that define important research needs for environmental chemistry and ecotoxicology:

1. The chemicals registered under REACH include many former “existing chemicals” that are structurally (very) similar to acknowledged POPs (persistent organic pollutants) or PBT chemicals (chemicals that are persistent, bioaccumulative, toxic). Accordingly, these “emerging chemicals” share hazardous properties such as high persistence and bioaccumulation potential with the structurally related POPs and PBT chemicals. Examples are brominated aromatic substances placed on the market as replacements of polybrominated diphenyl ethers (PBDEs used as flame retardants; one replacement is decabromodiphenyl ethane, see below) and a large group of poly- and perfluorinated alkyl substances (PFASs) placed on the market as replacements of the so-called long-chain PFASs such as PFOA or PFOS that were used, among others, in impregnating agents. These are cases of *incremental substitution* or *regrettable substitution*. Environmental chemists and ecotoxicologists need to use their extensive knowledge on legacy POPs and PBT substances in order to demonstrate, as quickly as possible, the environmental and health hazards associated with these “new” chemical products. Otherwise, the problems associated with the hazardous chemicals that have been banned (here: PBDEs, long-chain PFASs) will occur again and will then be perpetuated for many years and decades [8].
2. An unknown, but probably high number of these former existing chemicals that are placed on the market now as replacements of hazardous substances are still very poorly characterized. This is obvious from the data contained in the ECHA database, and the database suffers from a serious problem of insufficient data quality. A striking example is the brominated flame retardant DBDPE (CAS no. 84852-53-9), which has been registered with a very high volume of 10,000–100,000 t/year. For the octanol–water partition coefficient (log *K*_ow_) of this substance, the database shows a value of log *K*_ow_ = 3.55, which is too low by several log units, which is caused by a measurement error. The actual log *K*_ow_ of DBDPE is on the order of log *K*_ow_ = 11 [9]. This is an extreme case, but there are many more substances in the database for which erroneous data have been submitted in the registration dossiers. A systematic chemical and toxicological assessment of these data is urgently needed, but the methods and procedures for that are not yet in place. This complex evaluation of a vast amount of data requires substantial experience in physical chemistry, environmental chemistry, toxicology, and ecotoxicology.

## Conclusions on the demand for environmental chemistry and ecotoxicology

There is a serious misconception that needs to be rectified, namely that a problem has been solved as soon as it is covered by legislation. A regulation entering into force, such as REACH or the Water Framework Directive or the Stockholm Convention on POPs, does not indicate that the job has been done and that no more work will be needed. On the contrary, it marks the beginning of a period of increased demand for work: when a regulation is in place, this implies an obligation to establish the empirical basis that will make it possible to effectively implement and enforce the regulation. This means that empirical findings and data need to be generated, in-depth investigations to be carried out, and the state-of-affairs to be documented, often in considerable detail. Importantly, this goes beyond the routine work, but also includes long-term tasks such as the development of methods for sampling and data generation, methods for data interpretation, and transfer of all these methods to users in authorities and contract laboratories. All these elements will then form the empirical and conceptual foundations that need to be in place for a meaningful implementation of the legislation and, subsequently, its effectiveness evaluation. The problem of data availability and data quality under REACH is a case in point.

The two examples presented above demonstrate that the demand for research in environmental chemistry and ecotoxicology is caused by unresolved *old* problems such as the emissions and environmental and health impacts of PCBs, but also by many *new* issues such as the incremental substitution of hazardous chemicals under REACH or the monitoring of POPs that is a long-term obligation under the Stockholm Convention. For example, Wöhrnschimmel et al. [10] have shown that many more years of data generation will be needed before the effectiveness of the Stockholm Convention can be assessed. One would expect that the wide range of important and complex tasks for environmental chemistry and ecotoxicology would have helped to firmly establish these fields at universities. However, this is not the case and the ongoing cut-back on positions and resources for environmental chemistry and ecotoxicology is short-sighted and irresponsible. The underlying reasons for this situation may be manifold; to some extent, it could simply be lack of awareness and/or preferences for other, more “modern”, topics among the decision makers in universities. Another reason may be that environmental chemistry and ecotoxicology are perceived as fields of applied research without “true” academic relevance in comparison to well-established fields such as organic chemistry or booming areas such as material sciences.

## Academic standing of environmental chemistry and ecotoxicology

To evaluate the academic productivity of a field of research, two questions can be asked: (i) are new methods that are genuine to the field developed on the basis of ongoing research, i.e., is improving the methods, techniques, and tools a component of active and ongoing research in the field? (ii) Are the problems and questions investigated in the field continuously refined and are new questions and research objectives derived from the insights gained?

A closer inspection of our fields shows that both requirements are fulfilled for environmental chemistry and ecotoxicology. Obviously, there have been extraordinary improvements of analytical methods, but also a multitude of environmental factors that govern the environmental fate of chemicals, the many impacts of anthropogenic chemicals on environmental and human health, and the emission sources of many types of chemicals released to the environment are increasingly better understood, mechanistically characterized, and assembled as the elements of a big picture. However, as scientists in these fields, we have to point out the productivity of our research more explicitly. These discussions need to reach the decision makers in academic institutions.

## What is to be done?

First, a real danger to environmental chemistry and ecotoxicology is that they may be perceived (and presented) as fields where lists of routine tasks are worked down. If this happens, it will eliminate environmental chemistry and ecotoxicology as academic subjects. To confront this danger, the great demand for research and teaching in environmental chemistry and ecotoxicology and their academic productivity need to be pointed out explicitly in discussions in university committees tasked with priority setting—when positions, curricula, equipment, laboratory space, and financial resources are to be assigned and overall research priorities are to be determined.

Second, in addition to applied research and practical work, environmental chemistry and ecotoxicology have always had a strong component of basic research. Basic research is an essential part of these fields, and practical applications of methods and tools by authorities and industry are only possible because there is basic research that develops these methods and contributes to the scoping of problems and identifying relevant questions and tasks. Therefore, environmental chemistry and ecotoxicology need to be rooted in academic science, along with sufficient equipment, resources, and prospects for development. Environmental chemistry and ecotoxicology investigate a complex set of societally relevant issues, and there are many open and pressing problems related to the use of chemicals that have not been solved. As long as so many chemicals are present in so many technical applications and consumer products—which is considered a desirable aspect of modern societies—environmental chemistry and ecotoxicology are absolutely essential as the fields that help identify and understand the risks associated with the ever increasing use of chemicals.

Third, the environmental and health impacts of chemical pollution are not less important than other environmental impacts. Chemical pollution is one of the several globally relevant impacts as pointed out by Rockström et al. [11], who have identified nine impacts of global importance, ranging from climate change to chemical pollution, and they emphasize that these impacts do not act independently, but often reinforce one another. Recently, Rockström [12] stated:

“Among these nine there are three that have kind of come out as being the fundamental endgame of how all the planetary boundaries operate, and the number one is biodiversity. (…) The second fundamental boundary is climate change. (…) And the third of the big three is what we call “novel entities”. The totally man-made boundary. It has nothing to do with anything that the planet has ever experienced before, and it is our invention of chemicals, compounds, that are alien to nature like persistent organic pollutants (…).”

The call for a strong environmental chemistry and ecotoxicology could not be clearer.
